# Depth Plane Separation Affects Both Lightness Contrast and Assimilation

**DOI:** 10.3389/fpsyg.2020.02114

**Published:** 2020-09-01

**Authors:** Alessandro Soranzo, Steph Acaster, Naira Taroyan, John Reidy

**Affiliations:** Department of Psychology, Sociology and Politics, Faculty of Social Sciences and Humanities, Sheffield Hallam University, Sheffield, United Kingdom

**Keywords:** lightness contrast, lightness assimilation, belongingness, layer theories, anchoring theory

## Abstract

Lightness contrast and assimilation are two opposite phenomena: contrast occurs when a gray target perceptually acquires a complementary color than the bordering, inducing, surfaces; assimilation is when a gray target perceptually acquires the same color component as the inducers. Previous research has shown that both phenomena are affected by the manipulation of depth between the inducers and target. However, different results have been reported; it is not clear whether contrast persists when inducers are non-coplanar with the target. Previous studies differ for the spatial configuration of the stimuli and the technique adopted to manipulate depth. The aim of this research was to measure the effects of manipulating the depth between inducers and target in comparable conditions. Results show that contrast persists, but largely reduces, after depth manipulation while assimilation reverses to contrast. Furthermore, interesting asymmetries between white and black inducers emerged with white inducers favoring contrast and black inducers favoring assimilation. These results provide further evidence that high-level processes of visual processing are involved in both phenomena, with important consequences for lightness theories.

## Introduction

**Figure 1:**
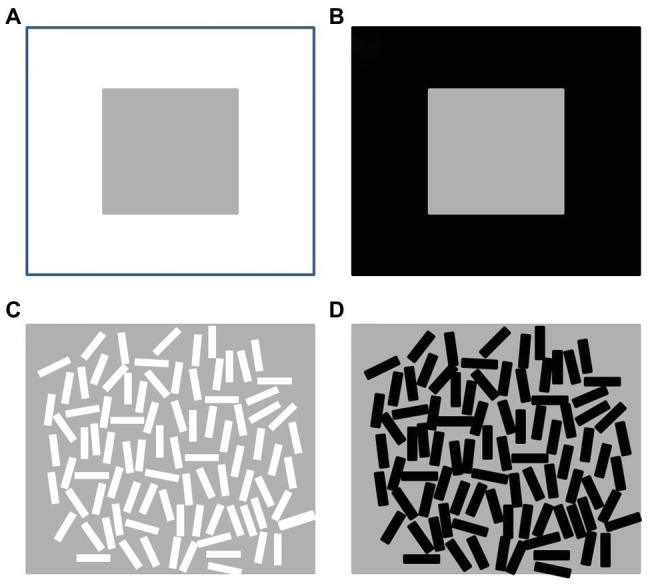
Lightness contrast (top row) and lightness assimilation (bottom row). Top row: the gray surrounded by white **(A)** is perceived to be darker than an equivalent gray surrounded by black **(B)**. Bottom row: the gray area with small white elements on top **(C)** is perceived to be lighter than an equivalent gray with black elements **(D)**.

The phenomena of lightness contrast and assimilation show that the color of a surface depends on the context within which it is seen. Contrast is the phenomenon by which a gray surface appears darker when it borders a light surface, and lighter when bordering a dark surface (see Figure 1, top row). This is, perhaps, one of the oldest and most studied phenomena in visual perception, having been described and investigated for over 2 millennia (Kingdom, 1999; see also Wade, 1996). Assimilation has received less attention from scholars; it has captured their interests only since the 19th century with the pioneering work of Chevreul (1839) and Von Bezold (1876). Assimilation occurs when a gray surface appears lighter when bordering a light surface, and darker when bordering a dark surface (see Figure 1, bottom row). Given that assimilation can be thought of as an effect which goes in the opposite direction to that of contrast, the relationship between contrast and assimilation presents an intriguing paradox, whereby the same surfaces produce opposite percepts under different conditions.

The relationship between contrast and assimilation was also described as paradoxical by Kanizsa (1979), particularly considering the presentation of contrast‐ and assimilation-eliciting stimuli simultaneously. Although in contrast, the gray target appears darker with white than with black inducers (Figure 1, A < B)^1^, and in assimilation, the target appears lighter with white than with black inducers (C > D), observers stated that the two gray targets neighboring white are equal to one another (A = C); likewise, the two targets neighboring black are equal to one another (B = D). Following this result, Kanizsa questioned whether contrast and assimilation exist as two distinct phenomena arising from different mechanisms or whether they share the same underlying process giving rise to different perceptions. The question of whether there are two completely distinct processes – one of contrast and one of assimilation – or whether both are manifestations of a single underlying process was previously raised by Helson (1963). He argued that, rather than being entirely separate and opposite phenomena, contrast and assimilation may actually be parts of a “continuum” of lightness phenomena, which includes a region in which neither contrast nor assimilation occurs. Helson and his collaborators (Helson and Rohles, 1959; Helson and Joy, 1962; Helson, 1963) noticed that when white and black striations are superimposed onto the same gray target, the gray appeared lighter for the white stripes and darker for the black stripes. However, the lightness in each case depended on the width, spacing, and color of the stripes. Thin lines with equally thin interspaces are optimal for eliciting assimilation, while thicker lines and larger separations elicit contrast. In other words, a spatial frequency change of the inducers generated a shift from one phenomenon to the other. Helson (1964) hypothesized that fine lines result in a summation process, producing assimilation, whereas coarse lines result in inhibition, producing contrast. He proposes that the two phenomena lie on a single continuum with a zone of neutrality, wherein there is neither effect.

The color of the inducers also seems to play a relevant role. While assimilation is described as an effect which goes in the opposite direction to contrast, there is some evidence to suggest that the difference between the gray with white and black inducers is a relative difference. De Weert and Spillman (1995) reported that in assimilation displays, the targets with either black or white inducers are always judged to be darker than a baseline. This would mean that white inducers always generate contrast effects, even in displays designed to generate assimilation.

Another important factor that can cause one effect to prevail over the other is the spatial relationship between target and inducers. As shown by Helson (1964), in coplanar displays, contrast effects emerge more clearly when the gray target is enclosed by the inducers while assimilation effects emerge more clearly when it is the inducer’s area, which is enclosed by the target (although a fascinating exception is offered by De Weert and Spillman, 1995).

Like Kanizsa and Helson, many researchers have been interested in the type of processing underlying contrast and assimilation, and several suggestions have been put forward to explain the phenomena. From one perspective, it has been suggested that a shift from contrast to assimilation is caused by bottom-up, “lower-order” mechanisms of visual processing. According to this view, assimilation is based on local averaging of luminance within the large receptive fields of neurons. Specifically, it has been proposed that a neuronal spatial integration (Hurvich and Jameson, 1966, 1974; DeValois and DeValois, 1975; Jameson and Hurvich, 1975) or weighted averaging (Reid and Shapley, 1988) occurs within receptive field centers. The Oriented Difference of Gaussians (ODOG; Blakeslee and McCourt, 1999, 2004) or its earlier non-oriented DOG version (Kingdom et al., 1997) models can be mentioned within this context. These models assume that lightness is encoded by banks of spatial filters whose receptive fields perform band-pass filtering of the spatial distribution of luminance in an image (Kingdom et al., 1997). These filters have a center-surround organization, such that the luminance in the surround inhibits the center response to a target. The center-surround structure of the filters account for the contrast effects, while assimilation arises from local averaging of intensity within large receptive field centers of the lowest frequency-tuned spatial filters, which encompass the inducers entirely.

A second perspective proposes that contrast and assimilation can be attributed to different levels of processing. Anchoring theory (Gilchrist et al., 1999), for example, gives an account of the way in which mid-level processes, including perceptual grouping-based frameworks, can account for contrast. Assimilation, on the other hand, is attributed to a “relatively low-level kind of space-averaged luminance” mechanism (Gilchrist et al., 1999, p. 802) and, therefore, explicitly not accounted by the theory.

Anchoring theory is probably the most representative theory within the framework approach, which includes theories that feature frames of reference but are also more rough and ready (Soranzo and Gilchrist, 2019). In contrast to the framework approach, the layers approach proposes that higher level of visual processing, such as figure/ground segmentation (Musatti, 1931, 1953; Festinger et al., 1970; Soranzo and Agostini, 2006a), or observer expertise (Kanizsa, 1979) can cause the shift between contrast and assimilation. Evidence that processes beyond retinal stimulation are involved in the assimilation phenomenon has been provided by, for example, de Weert and van Kruysbergen (1997), Soranzo et al. (2007), and Soranzo et al. (2010). de Weert and van Kruysbergen (1997) observed, by means of a stereogram to stratify the figure elements, that when some red and green disk-shaped-inducers are painted on a homogeneous white target-background, the latter appears reddish if the green inducers are perceived in a different plane, closer to the observer. Vice versa, the same target-background appears greenish when the red disks are those which appear closer to the observer (see de Weert and van Kruysbergen, 1997). This suggested that depth separation between surfaces may affect assimilation.

In the display of de Weert and van Kruysbergen (1997), however, the green and red disks (inducers) were both present on top of the white target at the same time. Because of this, it could be argued that the greenish and reddish appearance of the white target was caused by a contrast effect of the separated disks instead of an assimilation effect of the coplanar ones. In other words, in those conditions, it was not possible to distinguish whether the reddish and greenish appearance of the white target was caused by assimilation of the apparent coplanar disks, by contrast of the separated disks, or by both processes operating simultaneously. To control for this, Soranzo et al. (2010) tested separately the two types of inducers. Using a stereoscopic technique to manipulate the distance between high spatial frequency inducers and the target – that is, a stimulus configuration that elicits assimilation when inducers and target are coplanar, the authors found that in non-coplanar view, contrast rather than assimilation emerges. This result supports the importance for both contrast and assimilation of further processes than just the retinal stimulation because the retinal stimulation was practically equivalent in both coplanar and non-coplanar conditions.

Determining the effect that depth separation between surfaces has upon lightness perception is important for an understanding of the types of processing underlying phenomena such as lightness contrast and assimilation. Previously, it has been suggested that if there is no difference between the lightness of a surface in a coplanar display and the same surface in a retinally-equal, non-coplanar display, then, lightness processing must occur at a lower level of processing, prior to the processing of depth (Julesz, 1971; Gibbs and Lawson, 1974). Conversely then, an effect of depth separation on lightness perception suggests the involvement of higher-level processing (Gogel and Mershon, 1969; Mershon, 1972; Gilchrist, 1977; Soranzo et al., 2010).

Previous studies have investigated the effects of depth separation with stimuli which, in their coplanar counterpart, produced either contrast (Wolff, 1933; Gibbs and Lawson, 1974; Morikawa and Papathomas, 2002; Menshikova, 2013; Fujimoto and Ashida, 2015) or assimilation (Soranzo et al., 2010); not both. In addition, there is no agreement on the role of depth. Some findings show contrast effects persisting (e.g., Gibbs and Lawson, 1974) or even enhancing with depth manipulation (Fujimoto and Ashida, 2015); others report a reduction or elimination of contrast in non-coplanar conditions (Wolff, 1933); and others report a “shift” from assimilation in coplanar conditions to contrast in non-coplanar conditions (Soranzo et al., 2010). Different findings which manipulated depth, however, emerged in studies, which (a) used different displays types (either contrast or assimilation but not both in the same study) and (b) used different methods to manipulate depth (either actual or stereoscopic).

The aim of the current study is to investigate the role of actual depth on the lightness of the target in configurations that – when coplanar with the inducers – *clearly* elicit contrast or assimilation. Therefore, stimuli were constructed from paper and their configurations were designed in a way that, when coplanar, they elicit either contrast or assimilation as unambiguously as possible. Therefore, the experimental design included two levels of the spatial configuration variable: an “assimilation-eliciting” stimulus (high spatial frequency inducers enclosed within the target area) and a “contrast-eliciting” stimulus (low spatial frequency inducers enclosing the target area). The second independent variable was the color of the inducers (white and black). And finally, the third variable was the distance between the inducers and target (coplanar and non-coplanar, target 28 cm behind the inducers).

## Materials and Methods

### Participants

Twenty participants (15 females; mean age = 26.6), with no prior knowledge of the experiment, were recruited from Sheffield Hallam University. The sole pre-requisite for participation was that participants had normal (or corrected-to-normal) vision. The sample size was indicated by an *a priori* power analysis with α = 0.05, power = 0.90, and effect size *f* = 0.25.

The experiment was approved by the ethics committee of Sheffield Hallam University (Ref nr: AM/SW/42-ACA) and was conducted in accordance with the declaration of Helsinki (2008).

### Experimental Design and Stimuli

**Figure 2:**
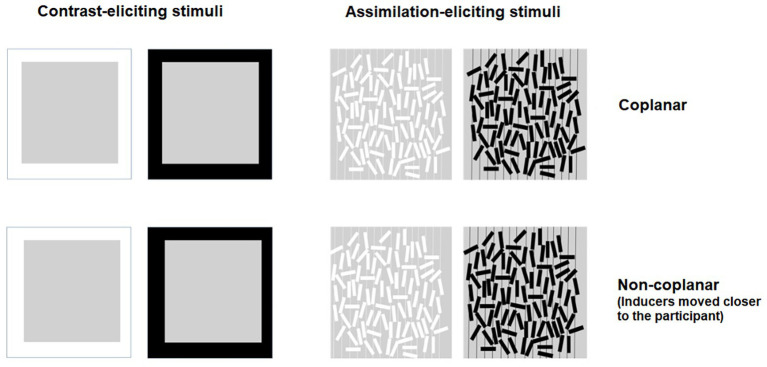
Sketch of the experimental stimuli.

The experimental design consisted of three independent variables: color of the inducers (white and black), stimulus configuration (“contrast-eliciting” and “assimilation-eliciting”), and inducers’ depth (“coplanar” and “non-coplanar”); see Figure 2. The eight conditions were presented in a 2 × 2 × 2 within-participants design. The dependent variable was the rated lightness of the gray target.

**Figure 3:**
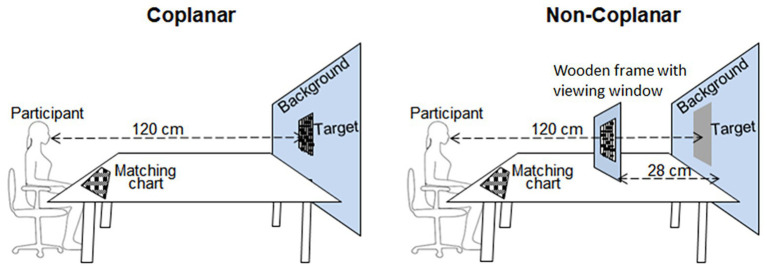
Sketch of the coplanar and non-coplanar conditions (see text for details).

The stimuli were constructed from paper, as follows. In a lab, homogeneously illuminated by a white neon lamp, a gray piece of paper (Munsell 5.6; 15.43 cd/m^2^) served as the target surface (see Figure 3). The target was visible through a square viewing window (10.4 × 10.4 cm) cut on a larger blue background that covered the entire visual field (see Figure 3). To minimize any potential inducing effect of the background, its reflectance was chosen to get approximately the same luminance as the target (32 cd/m^2^). Participants were seated 120 cm from the target.

For the four coplanar conditions, the inducers were glued onto the target; while for the non-coplanar conditions, inducers were cut (using a laser-cut printer which allowed for precise cuts) from white or black papers and suspended from a wooden frame. The frame was placed at a distance of 28 cm in front of the target, so that the gray target was visible at a distance behind the inducers.

For the coplanar conditions, inducers were formed, as follows. For the “contrast-eliciting” configuration, the gray target surface was visible through a square (8 × 8 cm; 3.82 × 3.82 deg.) that was surrounded by a frame-shaped inducer (width 2.3 cm; 1.1 deg.) which extended from the outside edge of the visible gray area to the inside edge of the viewing window. The “assimilation-eliciting” configuration consisted of 88 small rectangles (1.2 × 0.3 cm; 0.57 × 0.14 deg.) distributed across 15 thin lines (this was necessary to make the non-coplanar comparable, see below). The total area of the inducers was therefore the same for both the contrast and assimilation eliciting conditions (31 cm^2^). The color of the inducers was also the same for both the assimilation or contrast eliciting configurations: Munsell 9.5 (54.2 cd/m^2^) for the white inducers and Munsell 2.5 (3.4 cd/m^2^) for the black inducers.

For the non-coplanar conditions, the inducers were laser-cut from papers having the same reflectance as in the coplanar conditions (Munsell 9.5 for the white and Munsell 2.5 for the black inducer conditions), such that the small rectangles and the lines suspending the rectangles were a continuous surface of the same piece of paper. To the non-coplanar conditions comparable to the coplanar conditions, the size of the inducers in the non-coplanar conditions was slightly reduced: for the contrast eliciting conditions, the frame-shaped inducer width was 1.77 cm (1.1 deg.); for the assimilation-eliciting configuration, the 88 small rectangles were 0.92 × 0.23 cm; 0.57 × 0.14 deg.). In this way, both the visual angle subtended by the inducers and the total visible area of the gray target were the same in all conditions.

Alongside these stimuli, one of 12 matching charts was presented. For each trial, a stimuli and a chart were selected at random. Each chart contained 12 achromatic patches, ranging in equal logarithmic steps from 3.6 to 9.1 Munsell. luminance values (in cd/m^2^) of the patches in each chart were: 6.05, 8.01, 11, 12.02, 15.43 (target), 19.8, 24.1, 28.11, 33.89, 39, 44.7, and 51.11.

**Figure 4:**
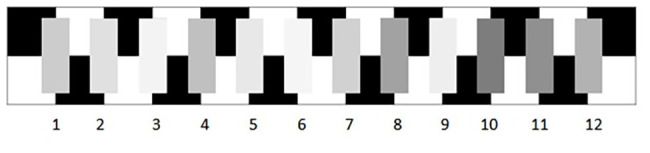
Example matching chart.

The patches within each chart were arranged in a random order, so that the same patch was indicated by a different number in each chart. To minimize potential effects of the background, the matching patches were presented on a black and white checkerboard background (i.e., the same colors of the inducers). Furthermore, to avoid potential confounds of the illumination, both the charts and the stimuli were illuminated by equal light sources placed at the same distance. Figure 4 shows one example of a matching chart.

### Procedure

Participants were required to read through an information sheet and give written informed consent before commencing the study. They were instructed that the researcher would be changing elements of the display and that their task was to choose the patch on the matching chart which “looks as though it is the same gray paper as the target was cut from”. Between trials, participants were asked to turn away from the display to allow the researcher to change the inducers, frame position, and matching chart, according to a randomized list. Participants then turned back to face the display and verbally gave the number corresponding to the gray they wished to choose from the matching chart. There was no time limit for participants to decide on a response. Each participant responded to two repetitions of each stimulus presented in a random order.

## Results

Consistent with Weber-Fechner law (Fechner, 1860/1912), each match was converted into the logarithm of the luminance value corresponding to the chosen patch. For each of the responses, the log luminance ratio between the baseline (15.43 cd/m^2^) and the participant’s selected value was calculated.

Each participant responded to two stimuli per condition, so for each of the eight conditions, a mean response per participant was calculated:

$$\begin{array}{l} \text{Transformed data}=\text{Mean}\\ \left[\text{log}\left(\text{luminance of matching patch}/\text{target}\right)\right] \end{array}$$

**Figure 5:**
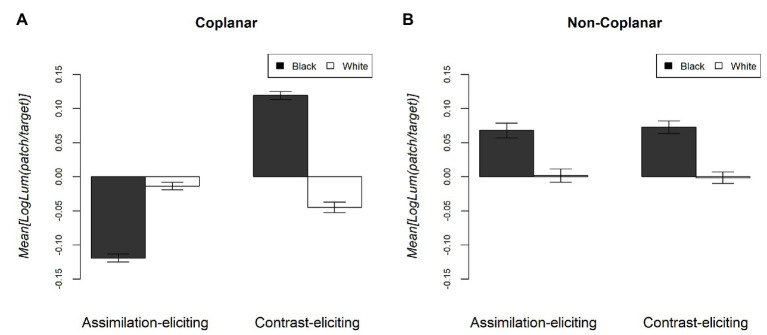
Mean difference between baseline and perceived luminance of the target (log luminance) for each condition. Zero represents the baseline value (measured luminance of the target). Positive values indicate that the match was perceived to be lighter than the target and negative values indicate that the match was perceived to be darker. Error bars represent standard errors. **(A)** shows the results for the Coplanar conditions whilst **(B)** indicates the results for the Non-Coplanar conditions.

Two scores provided by two different participants in two experimental conditions, being more than three standard deviations from the mean of the respective condition means, were transformed to the next-most extreme score within the same condition (Tabachnick and Fidell, 2014). The mean difference between the participants’ selected values and the measured target luminance value for each condition are shown in Figure 5, with zero representing the “baseline” measured luminance value of the target. Positive values represent a perception of the target that is lighter than the baseline, whereas negative values represent a perception of the target that is darker than the baseline.

The transformed data were analyzed using a 2 × 2 × 2 repeated-measures analysis of variance (ANOVA), showing a significant three-way interaction between color of the inducers, stimulus configuration, and inducers’ depth [*F*(1,19) = 46.61, *p* < 0.001, η*_p_*^2^ = 0.71]. This interaction was then further explored by analyzing the data separately for coplanar conditions and non-coplanar conditions, using two 2 × 2 ANOVAs.

In the coplanar conditions, the two-way interaction between stimulus configuration and color of the inducers was significant [*F*(1,19) = 92.5, *p* < 0.001, η*_p_*^2^ = 0.83]. *Post hoc t*-tests showed that, for the “assimilation-eliciting” conditions, targets were judged to be darker with black inducers than with white inducers [*t*(19) = 6.97, *p* < 0.001], representing an assimilation effect^2^. For the “contrast-eliciting” conditions, targets were judged to be *lighter* with black inducers than with white inducers [*t*(19) = 8.5, *p* < 0.001], representing a contrast effect. In this case, the target deviates more from the baseline, in absolute values, with black inducers than with white ones.

In the non-coplanar conditions, there was no significant two-way interaction between stimulus configuration and color of the inducers [*F*(1,19) = 0.172, *p* = 0.683, η*_p_*^2^ = 0.01]. There was no significant main effect of stimulus configuration [*F*(1,19) = 0.007, *p* = 0.935, η*_p_*^2^ < 0.001], but there was a significant main effect of color of inducers [*F*(1,19) = 0.098, *p* = 0.003, η*_p_*^2^ = 0.371]. For both types of stimulus configuration, targets were judged lighter with black inducers than with white inducers, thus representing an overall contrast effect. However, white inducers did not make the target deviate significantly from the baseline.

The effects of distance were further measured by a series of *post hoc t*-tests. They showed that distance significantly reduces the effects of contrast when the inducers were black [t(19) = 3.02; *p* < 0.01]; a similar effect was registered when the inducers were white [t(19) = 1.94; *p* = 0.067] although this test did not reach statistically significance. Furthermore, assimilation effects also reduced with distance when the inducers were black [t(19) = 8.67; *p* < 0.01] but no when the inducers were white [t(19) = 0.78; *p* = 0.44].

## Discussion

The experiment shows that the pattern of results obtained in the coplanar conditions did not hold once the inducers and target underwent depth separation, into the non-coplanar conditions. In coplanar conditions, the color of the inducers had a different effect on the lightness of the target, depending on the stimulus configuration: in contrast-eliciting stimuli, the target was judged to be lighter with black inducers than with white inducers; in assimilation-eliciting stimuli, targets were judged to be lighter with white inducers than with black inducers. However, in non-coplanar conditions, stimulus configuration did not appear to influence the direction of the effect: for both configurations, targets were perceived darker with white than with black inducers. The strength and symmetries of the both effects were, however, affected by depth, as it will be discussed shortly.

### Coplanarity Versus Depth Separation

Depth separation appears to have a different effect depending upon the stimulus configuration. The assimilation effect emerging with assimilation-eliciting stimuli is reversed by depth separation; it becomes contrast. However, contrast effect emerging with contrast-eliciting stimuli persists even with depth separation, although its magnitude is reduced (see Economou et al., 2015). The results are consistent with the findings in which assimilation-eliciting stimuli generate assimilation effects only in coplanar conditions, but they generate contrast effects in non-coplanar conditions (Soranzo et al., 2010).

It seems, therefore, that the stimulus configuration, specifically, the spatial frequency of the inducers together with their spatial relationship with the target, plays a crucial role on the lightness of the target. Considering the spatial relationship between inducers and target, depth separation can be viewed as a manipulation which “disrupts” the coplanar contrast and assimilation effects. Thus, an observation can be made that the effect of depth separation depends on the “starting point”, i.e., the type of effect produced in the coplanar condition, which in turn depends on the spatial frequency of inducers and their spatial relationship with the target. Given that contrast effects emerged in all the non-coplanar conditions, even for the assimilation-eliciting stimuli, it can also be argued that assimilation emerging in coplanar conditions is disrupted by depth separation to a greater extent than is contrast. This suggests that, perhaps, assimilation is the less robust of the two phenomena, as contrast continues to occur in non-coplanar conditions. In addition, in line with the findings of De Weert and Spillman (1995), in the assimilation-eliciting displays with both white and black inducers, targets are always judged to be darker than the baseline. Technically, this would make the effect with white inducers a (small) contrast effect also in the coplanar condition as the target lightness is perceived further away from the inducer color. Assimilation is therefore only a relative phenomenon. Given that, relative to the target with black inducers, the target with white inducers is perceived lighter, then we call this effect assimilation.

These suggestions are counter in some ways with the observation of DeValois and DeValois (1975) that assimilation might be more common in nature than contrast. It seems improbable that a weaker phenomenon such as assimilation occurs more often than the more persistent one of contrast.

### The Role of the Method Used to Manipulate Depth

As mentioned in the introduction, there are important inconsistencies in the literature on the effects of depth separation between inducers and target with contrast-eliciting displays. Analyzing the results of the current work in comparison to previous outcomes, it seems that the apparatus and technique used to manipulate depth play an important role on the lightness of the target. To sum up, the study of Wolff (1933) and the current study found that contrast reduces with depth, while the studies of Julesz (1971), Gibbs and Lawson (1974), Fujimoto and Ashida (2015), and Menshikova (2013) found that contrast persists or is even enhanced.

These inconsistencies may be due to the method adopted to manipulate depth^3^. While studies which found that contrast persists or even is enhanced used stereoscopic depth, those studies which found that contrast reduces or disappears used real depth. In this regard, Gilchrist (2006) suggested that, “the additional cues present in Wolff’s study like accretion and deletion at target edges due to observer motion might account for the different results” (p. 278). Future works on stereoscopic depth might usefully test binocular vs. monocular vision. The effects of depth caused by ocular disparity may then be clearer.

In addition, those that found that contrast persists – or is enhanced – presented the stimuli on a computer monitor. Agostini and Bruno (1996) reported that contrast effects are larger on the computer screen rather than on paper. It is therefore suggested that the larger contrast effect found in computer presentations of stimuli in combination with the different methods used to add depth between target and inducers might explain the inconsistencies.

### Asymmetries Associated With Color of Inducers

The results demonstrate that the effects of inducer color are not “symmetrical” when comparing equivalent conditions, which differ only with respect to this variable. For the contrast-eliciting conditions, the contrast effect registered with black inducers is larger than that with white inducers. The same asymmetry persists in the non-coplanar conditions. The overall magnitude of contrast, however, was reduced in the non-coplanar condition compared to the coplanar condition^4^.

In our experiment, assimilation-eliciting stimuli generated assimilation effects only with black inducers. This is consistent with previous research which suggested that black inducers favor assimilation more than white inducers (Beck, 1966; Agostini et al., 2001; Murgia et al., 2016). It could be hypothesized that white inducers need to be smaller and/or more numerous than black inducers to generate assimilation. Although this possibility would be interesting to examine, it does not explain the asymmetry that emerged here: inducers of the same size and number generate assimilation only when they are black, not white.

In addition, assimilation-eliciting stimuli generate assimilation only in coplanar conditions, while they generate contrast effects when depth between inducers and target is added. This result is congruent with the findings of Soranzo et al. (2010), who used a stereoscopic technique to manipulate depth, while here we used a real depth manipulation. It seems therefore that regardless of the type of manipulation of depth, assimilation emerges only in coplanar conditions and only with inducers darker than the target.

As can be seen from Figures 5A,B, the target deviates from the baseline, in absolute values, more with black inducers than with white inducers. In fact, in coplanar conditions, the lightness of the target bordering white inducers deviates from the baseline just slightly (and always in the contrast direction, even with assimilation-eliciting configurations), while it practically does not deviate at all when depth is added. To explain this effect, we consider the gamut expansion effect suggested by Gilchrist et al. (1999) in the anchoring theory. According to the authors, in contrast-eliciting configurations, a target bordering white inducers undergoes a smaller, relative to a target bordering black inducers, but still significant deviation from the baseline. This is due to the tendency of the range of perceived grays in a region of visual stimuli (a framework) to expand when the luminance range is restricted. In our contrast-eliciting condition with white inducers, the luminance range in our stimuli was indeed restricted as there were just the gray target and the white inducers (this effect has been systematically investigated by Economou et al., 2007).

To account for the fact that the effects of gamut expansion did not occur when depth was added (see again Figure 5B), it can be advanced that the gamut expansion phenomenon involves only surfaces lying on the same depth plane.

### Implications for Lightness Theories

The findings that depth manipulation was found to affect both contrast and assimilation are difficult to interpret within the low level, lateral inhibition inspired models such as DOG and ODOG. In fact, the retinal stimulation, within the same eliciting conditions, was practically the same. The retinal stimulations and the corresponding spatial filtering analysis should have been the same between coplanar and non-coplanar conditions, but this was not the case. The asymmetries associated with the color of inducers are also difficult to be interpreted within these models. These findings can be accommodated within decomposition theories such as the framework and layer theories (see Soranzo et al., 2013; Soranzo and Gilchrist, 2019) as we will show here.

As mentioned in the introduction, the framework-based anchoring theory (Gilchrist et al., 1999) neglects assimilation and proposes that this phenomenon may occur at a lower level of the visual process than contrast. The current findings do not support this. As assimilation-eliciting stimuli generated contrast in non-coplanar conditions even though the retinal stimulation was similar to the coplanar condition, it seems that both phenomena occur at a level of visual processing beyond the retinal stimulation. However, the effects of area on perceived illumination can, at least partially, reconcile the anchoring theory with assimilation. In our assimilation-eliciting stimuli with white inducers, the lightest surface (the inducers) was not the larger area, as it was the gray target. Gilchrist and Soranzo (2019) suggested that in the conditions in which the largest area is not the lightest, enlarging the surface with the darker area “causes its lightness to increase and the perceived illumination to decrease” (p. 1470). In the non-coplanar condition, the area of the gray target perceptually increases as it is perceived to extend behind the white inducers. According to the anchoring theory, this perceptual extension of the target areas should increase its lightness, which is what we have found. It will be interesting to measure perceived illumination of these stimuli. According to the anchoring theory, depth separation of assimilation-eliciting stimuli with white inducers should reduce perceived illumination.

The same line of reasoning, however, cannot be adopted for the assimilation effects emerging with black inducers as the gray target is both the lightest and the largest area. In this case, the account offered by King (1988) seems more appropriate. King noted that a transition from contrast to assimilation occurs as a function of increasing “positional similarity” or closeness between targets and inducers (as in Helson and Joy, 1962), or as a function of increasing similarity in the color of the targets and inducers (as in Beck, 1966). King suggested that when perceptual belongingness^5^ produces a single perceptual unit, assimilation is favored; contrast instead occurs when inducers and target are perceived as two independent units. A similar explanation was offered by Musatti (1953), who advanced that assimilation occurs when the inducing elements are fragmented and dispersed within the target area, appearing as the texture of a *unitary whole*. In our assimilation-eliciting stimuli, the belongingness between the inducers and the target was strong only in the coplanar condition, while it was reduced when depth was added. When depth was added, assimilation did not emerge anymore. Many studies have, however, found that increasing belongingness between the inducers and the target elicits contrast (e.g., Benary, 1924; Agostini and Proffitt, 1993; Agostini and Galmonte, 2002). It can therefore be suggested that strengthening belongingness between inducers and target increases contrast only up to a certain point; when it becomes so “*extreme*” to generate the percept of a single unit (as it may have happened in our assimilation-eliciting stimuli, coplanar condition), contrast does not increase anymore. What happens to the lightness of the target when belongingness is *extreme* depends on the color of the inducers: when the inducers are darker than the target, *extreme* belongingness reverses contrast to assimilation; when the inducers are lighter, *extreme* belongingness reduces contrast, but it does not reverse it into assimilation.

To explain this differential effect of *extreme* belongingness, it can be assumed that both contrast and assimilation result from two competing processes: on the one side, anchoring processes (i.e., the tendency of the highest luminance and larger area to appear white) favor contrast; on the other side, *extreme* belongingness favors assimilation. In assimilation-eliciting stimuli, when the inducers are black, the tendency of the gray target to be perceived as white (because it is locally the lighter area) is overtaken by *extreme* belongingness; when the inducers are white, instead, it is the *extreme* belongingness to be overtaken by the more powerful anchor of the white inducers. In this latter case, the inducers are the lighter area globally, not only locally, hence their relatively stronger power as anchor. Further studies are needed to clarify whether extreme belongingness is a mediating factor in lightness perception.

### Conclusion

The findings of this study demonstrate that depth separation between inducers and target has an effect on lightness contrast and lightness assimilation even if the retinal image is similar. These results provide further evidence to suggest that high-level processes mechanisms are involved in both contrast and assimilation. These mechanisms may involve both anchoring processes, favoring contrast effects, and extreme belongingness, favoring assimilation effects.

## Data Availability Statement

The data are available at https://osf.io/5xp4z/.

## Ethics Statement

The experiment was approved by the ethics committee of Sheffield Hallam University (Ref nr: AM/SW/42-ACA). The participants provided their written informed consent to participate in this study.

## Author Contributions

AS designed the study and contributed to the writing up of the manuscript. SA and NT collected the data and contributed to the writing up of the manuscript. JR analyzed the data and contributed to the writing up of the manuscript. All authors contributed to the article and approved the submitted version.

### Conflict of Interest

The authors declare that the research was conducted in the absence of any commercial or financial relationships that could be construed as a potential conflict of interest.

## Footnotes

^1^To the benefit of clarity, we refer to the gray surface as the *target* and the bordering surfaces as the *inducers*.

^2^Technically, this would make the effect with white inducers a (small) contrast effect also in the coplanar condition. However, given that ‐ relative to the target with black inducers ‐ the target with white inducers is perceived *lighter*, this effect can be described as relative assimilation. This is congruent with the suggestion of De Weert and Spillman (1995) outlined in the introduction of this paper.

^3^Another difference among the different studies on the effects of depth on contrast eliciting stimuli is that depth was manipulated by moving either the target or the inducers closer to the participant. However, this does not seem to be a crucial factor. Indeed, in both the current study and the study of Fujimoto and Ashida (2015), the inducers were moved closer to the participants; nevertheless, the results are opposite.

^4^Wolff (1933) suggested that contrast disappears or reduces when inducers and targets are non-coplanar. It seems that contrast is quite a robust effect that does not totally disappear with depth manipulation.

^5^Perceptual belongingness refers to the processes that are responsible for determining how the part-whole structure of experienced perceptual objects is derived from the unstructured data in retinal images (Wertheimer, 1923/1939). See Soranzo and Agostini (2006b) for a review on the effects of perceptual belongingness on lightness.

## References

[ref36] Agostini T. Bruno N. (1996). Lightness contrast in CRT and paper-and-illuminant displays. Percept. Psychophys. 58, 250–258. 10.3758/BF03211878, PMID: 8838167

[ref1] Agostini T. Daris D. Galmonte A. (2001). Kanizsa’s paradox revisited. J. Vis. 1:424. 10.1167/1.3.424

[ref2] Agostini T. Galmonte A. (2002). Perceptual organisation overcomes the effects of local surround in determining simultaneous lightness contrast. Psychol. Sci. 13, 89–93. 10.1111/1467-9280.00417, PMID: 11892786

[ref3] Agostini T. Proffitt D. R. (1993). Perceptual organization evokes simultaneous lightness contrast. Perception 22, 263–272. 10.1068/p220263, PMID: 8316514

[ref4] Beck J. (1966). Contrast and assimilation in lightness judgments. Percept. Psychophys. 1, 342–344. 10.3758/BF03207403

[ref5] Benary W. (1924). Beobachtungen zu einem Experiment uber Helligkeitskontrast. Psychol. Forsch. 5, 131–142. 10.1007/BF00402398

[ref6] Blakeslee B. McCourt M. E. (1999). A multiscale spatial filtering account of the White effect, simultaneous brightness contrast and grating induction. Vis. Res. 39, 4361–4377. 10.1016/S0042-6989(99)00119-4, PMID: 10789430

[ref7] Blakeslee B. McCourt M. E. (2004). A unified model of brightness contrast and assimilation incorporating oriented multiscale spatial filtering and contrast normalization. Vis. Res. 44, 2483–2503. 10.1016/j.visres.2004.05.015, PMID: 15358084

[ref50] Chevreul M. E. (1839). De la lot du contraste simultane des couleurs (The principles of harmony and contrast of colors).

[ref8] De Weert C. M. Spillman L. (1995). Assimilation: asymmetry between brightness and darkness? Vis. Res. 35, 1413–1419. 10.1016/0042-6989(95)98721-K, PMID: 7645270

[ref52] de Weert C. M. van Kruysbergen N. A. (1997). Assimilation: central and peripheral effects. Perception 26, 1217–1224. 10.1068/p261217, PMID: 9604059

[ref9] DeValois R. L. DeValois K. K. (1975). “Neural coding of color” in Handbook of perception. Vol. 5 eds. Carterette E. C. Friedman M. P. (New York: Academic Press), 156–157.

[ref11] Economou E. Zdravkovic S. Gilchrist A. (2007). Anchoring versus spatial filtering accounts of simultaneous lightness contrast. J. Vis. 7, 1–15. 10.1167/7.12.2, PMID: 17997644

[ref12] Economou E. Zdravković S. Gilchrist A. (2015). Grouping factors and the reverse contrast illusion. Perception 44, 1383–1399. 10.1177/0301006615607118, PMID: 26562863

[ref13] Fechner G. T. (1860/1912). Elemente der Psychophysik. Liepzig: Breitkopf und Hartel.

[ref14] Festinger L. Coren S. Rivers G. (1970). The effect of attention on brightness contrast and assimilation. Am. J. Psychol. 83, 189–207. 10.2307/1421323, PMID: 5450896

[ref15] Fujimoto K. Ashida H. (2015). Asymmetric effects of stereoscopic depth on simultaneous lightness contrast. Perception 44:316.

[ref16] Gibbs T. Lawson R. B. (1974). Simultaneous brightness contrast in stereoscopic space. Vis. Res. 14, 983–987. 10.1016/0042-6989(74)90167-9, PMID: 4432398

[ref17] Gilchrist A. (1977). Perceived lightness depends on perceived spatial arrangement. Science 195, 185–187. 10.1126/science.831266, PMID: 831266

[ref53] Gilchrist A. (2006). Seeing black and white. Vol. 40 OUP USA.

[ref18] Gilchrist A. Kossyfidis C. Bonato F. Agostini T. Cataliotti J. Li X.. (1999). An anchoring theory of lightness perception. Psychol. Rev. 106, 795–834. 10.1037/0033-295X.106.4.795, PMID: 10560329

[ref19] Gilchrist A. Soranzo A. (2019). What is the relationship between lightness and perceived illumination. J. Exp. Psychol. Hum. Percept. Perform. 45, 1470–1483. 10.1037/xhp0000675, PMID: 31556684

[ref20] Gogel W. C. Mershon D. H. (1969). Depth adjacency in simultaneous contrast. Percept. Psychophys. 5, 13–17. 10.3758/BF03210471

[ref21] Helson H. (1963). Studies of anomolous contrast and assimilation. J. Opt. Soc. Am. 53, 179–184. 10.1364/JOSA.53.000179, PMID: 13953661

[ref22] Helson H. (1964). Adaptation-level theory. New York: Harper & Row, 282–292.

[ref23] Helson H. Joy V. L. (1962). Domains of lightness assimilation and contrast. Psychol. Beit. 6, 405–415.

[ref24] Helson H. Rohles F. G. (1959). A quantitative study of reversal of classical lightness contrast. Am. J. Psychol. 72, 530–538. 10.2307/1419494, PMID: 14400942

[ref25] Hurvich L. M. Jameson D. (1966). The perception of brightness and darkness. Boston, MA: Allyn & Bawn.

[ref26] Hurvich L. M. Jameson D. (1974). Opponent processes as a model of neural organization. Am. Psychol. 29, 88–102. 10.1037/h0035924, PMID: 4204851

[ref27] Jameson D. Hurvich L. M. (1975). From contrast to assimilation: in art and in the eye. Leonardo 8, 125–131. 10.2307/1572954

[ref28] Julesz B. (1971). Foundations of cyclopean perception. Chicago, IL: The University of Chicago Press.

[ref29] Kanizsa G. (1979). Organization in vision: Essays on gestalt perception. New York: Praeger Publishers.

[ref30] King D. L. (1988). Assimilation is due to one perceived whole and contrast is due to two perceived wholes. New Ideas Psychol. 6, 277–288. 10.1016/0732-118X(88)90039-6

[ref31] Kingdom F. (1999). Old wine in new bottles? Some thoughts on Logvinenko’s “lightness induction revisited”. Perception 28:929. 10664745 10.1068/p2808ed

[ref32] Kingdom F. A. A. McCourt M. E. Blakeslee B. (1997). In defence of “lateral inhibition” as the underlying cause of induced brightness phenomena: a reply to Spehar, Gilchrist and Arend. Vis. Res. 37, 1039–1044. 10.1016/s0042-6989(96)00258-1, PMID: 9196722

[ref33] Menshikova G. Y. (2013). An investigation of 3D images of the simultaneous-lightness-contrast illusion using a virtual-reality technique. Psychol. Russ. 6, 49–59. 10.11621/pir.2013.0305

[ref34] Mershon D. H. (1972). Relative contributions of depth and directional adjacency to simultaneous whiteness contrast. Vis. Res. 12, 969–979. 10.1016/0042-6989(72)90018-1, PMID: 5037712

[ref35] Morikawa K. Papathomas T. V. (2002). Influences of motion and depth on brightness induction: an illusory transparency effect? Perception 31, 1449–1457. 10.1068/p3439, PMID: 12916669

[ref37] Murgia M. Prpic V. Santoro I. Sors F. Agostini T. Galmonte A. (2016). Perceptual belongingness determines the direction of lightness induction depending on grouping stability and intentionality. Vis. Res. 126, 69–79. 10.1016/j.visres.2015.10.018, PMID: 27208582

[ref38] Musatti C. (1931). Forma e assimilazione. Arch. It. Psic. 9, 213–269.

[ref39] Musatti C. (1953). Ricerche sperimentali sopra la percezione cromatica. [Experimental research on chromatic perception]. Arch. It. Psic. Neur. e Psichiat. 14, 541–577. 13115131

[ref40] Reid R. C. Shapley R. (1988). Brightness induction by local contrast and the spatial dependence of assimilation. Vis. Res. 28, 115–132. 10.1016/0042-6989(88)90013-2, PMID: 3413989

[ref41] Soranzo A. Agostini T. (2006a). Photometric, geometric and perceptual factors in illumination-independent lightness constancy. Percept. Psychophys. 68, 102–113. 10.3758/bf03193660, PMID: 16617834

[ref42] Soranzo A. Agostini T. (2006b). Does perceptual belongingness affect lightness constancy? Perception 35, 185–192. 10.1068/p5342, PMID: 16583764

[ref43] Soranzo A. Galmonte A. Agostini T. (2010). Von Bezold assimilation effect reverses in stereoscopic conditions. Perception 39, 592–605. 10.1068/p6462, PMID: 20677697

[ref44] Soranzo A. Gilchrist A. (2019). Layer and framework theories of lightness. Atten. Percept. Psychophysiol. 81, 1179–1188. 10.3758/s13414-019-01736-1, PMID: 31044399 PMC6647447

[ref45] Soranzo A. Lugrin J. Z. Wilson C. (2013). The effects of belongingness on the simultaneous lightness contrast: a virtual reality study. Vis. Res. 86, 97–106. 10.1016/j.visres.2013.04.012, PMID: 23664881

[ref46] Soranzo A. Puppa N. D. Quinn G. (2007). The assimilation contrast shift phenomenon. Perception 36:82.

[ref47] Tabachnick B. G. Fidell L. S. (2014). Using multivariate statistics. Essex, UK: Pearson.

[ref48] Von Bezold W. (1876). The theory of color and its relation to art and art-industry. (Koehler S. R., trans.). Boston: L. Prang and Company.

[ref49] Wade N. J. (1996). Descriptions of visual phenomena from Aristotle to Wheatstone. Perception 25, 1137–1175. 10.1068/p251137, PMID: 9027920

[ref54] Wertheimer M. (1923/1939). “Untersuchungen zur Lehre von der Gestalt [Laws of organization in perceptual forms]” in A source book of gestalt psychology. ed. Ellis W. D. (London: Routledge & Kegan Paul), 301–350.

[ref51] Wolff W. (1933). Über die kontrasterregende Wirkung der transformierten Farben (concerning the contrast-causing effect of transformed colours). Psychol. Forsch. 18, 90–97. 10.1007/BF02409628

